# Global, national and regional prevalence, and associated factors of ocular trauma: A protocol for systematic review and meta-analysis

**DOI:** 10.1097/MD.0000000000021870

**Published:** 2021-09-21

**Authors:** Xiaoyan Bian, Shuang Xu, Yuli Song, Yuye Wang, Bin Zhao, Yifan Zhong, Lei Liu, Yuedong Hu

**Affiliations:** a Department of Ocular Surface, Baotou Chaoju Eye Hospital, Baotou; b Department of Library, China Medical University; c China Medical University; d YunCheng Eye Hospital, Yuncheng; e Department of Ophthalmology, The First Affiliated Hospital of China Medical University, Shenyang, China.

**Keywords:** ocular trauma, prevalence, risk factors, systematic review

## Abstract

**Background:**

Ocular trauma is a common eye disease and one of the main causes of blindness. There is a dearth of data on a summary and meta-analysis on the global epidemiology of the disease. Therefore, this systematic review protocol aims to propose the first systematic review and meta-analysis to synthesize existing evidence on the global prevalence and associated factors of ocular trauma worldwide.

**Methods:**

A systematic search will be performed according to the following databases: PubMed, Web of Science, Chinese National Knowledge Infrastructure (CNKI), Weipu, and Wanfang. Cross-sectional, case-control, and cohort studies reporting on the prevalence and risk factors of ocular trauma will be included. The primary outcome will be the prevalence in global, regional, and national ocular trauma. Study searching, data extraction, and quality evaluation will be performed by 2 reviewers, independently. Appropriate meta-analysis will then be used to pool studies. STATA software package v 12.0 (Stata Corporation, College Station, TX) and R (version 3.4.1; R Foundation for Statistical Computing, Vienna, Austria) software will be used for all statistical analyses.

**Results:**

This study will provide a high-quality synthesis to examine the prevalence and associated factors of ocular trauma worldwide. Furthermore, current study will project disease estimates in the next 50 years.

**Conclusion:**

This systematic review and meta-analysis will provide first evidence to evaluate the burden of ocular trauma in the general population.

**Ethics and dissemination:**

This systematic review and meta-analysis of randomized controlled trials does not require ethical recognition, and the results of this paper will be published in an open access, internationally influential academic journal.

**Trial registration number:**

CRD42020189166

## 1 Introduction

Ocular trauma is one of the most important causes of ocular morbidity and visual impairments. According to previous reports, ocular trauma is usually the third and fourth cause of binocular and monocular blindness, respectively. There are an estimated 55 million eye injuries occurring annually, of which 19 million have vision loss or blindness.^[1,2]^ Severe ocular trauma can lead to permanent visual impairment, as well as corneal, lens, or retinal complications.^[3]^ Generally, ocular trauma always occurs in childhoods, who are during critical period of growth, the health care impact can be significant.

Previously, prevalence rates of ocular trauma ranged from 14.4% up to 21.1% in Western countries, and people living these area with young age, male sex, and lower socioeconomic status, poor education levels, or engaged in labor-intensive occupations mostly have a high risk of ocular trauma.^[4–6]^ While in Asia, some population-based studies reported that the prevalence of ocular trauma was 4.4% in Singapore Chinese population, 3.6% in Chinese population in Beijing, and 2.1% in Handan, respectively.^[3,7,8]^ In Singapore, there is no association between occupation and ocular trauma.^[7]^ Herein, the prevalence and risk factors of ocular trauma vary from region to region, and the global evidence of these estimates on ocular trauma is rare.

Furthermore, we will perform a systematic review and meta-analysis to provide knowledge of the prevalence, and risk factors of ocular trauma informing public health and allow development of strategies to reduce the socioeconomic burden of ocular trauma worldwide.

## 2 Methods

This review is performed in accordance with the preferred reporting items for systematic review and meta-analysis (PRISMA) protocols guidelines.^[9]^

Furthermore, this protocol is registered on the International Prospective Register of Systematic Reviews numbered CRD42020189166.

The PRISMA for protocol checklist was shown in Table 1.

**Table 1:**
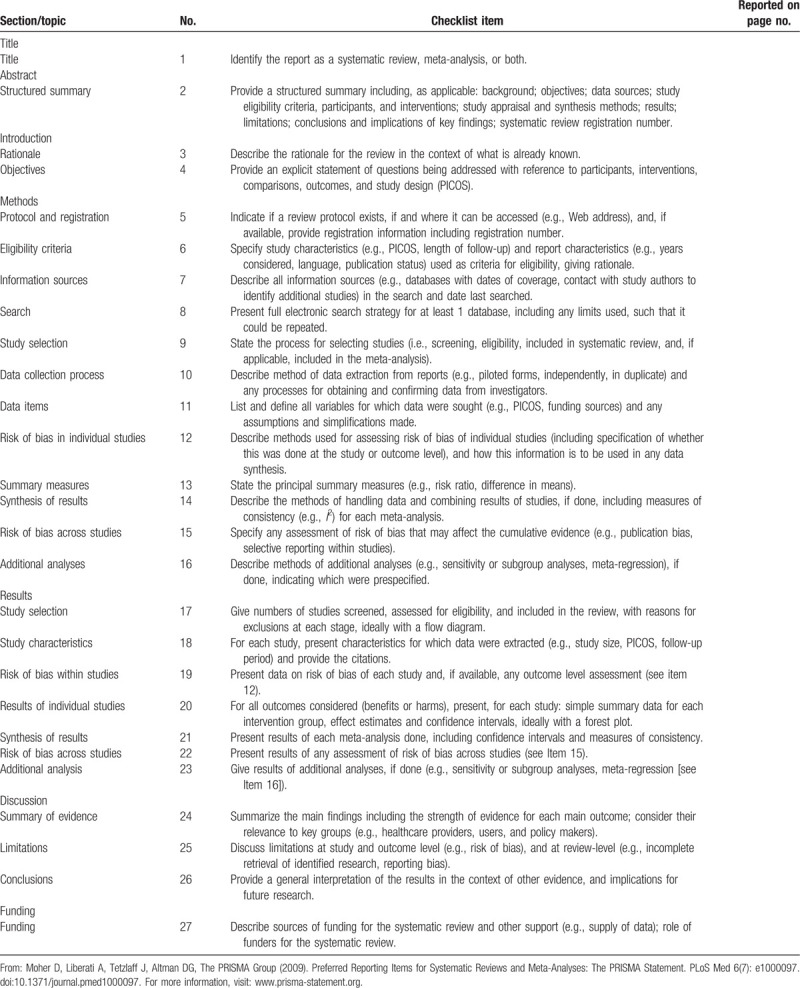
PRISMA 2009 checklist.

### 2.1 Search strange

**Figure 1:**
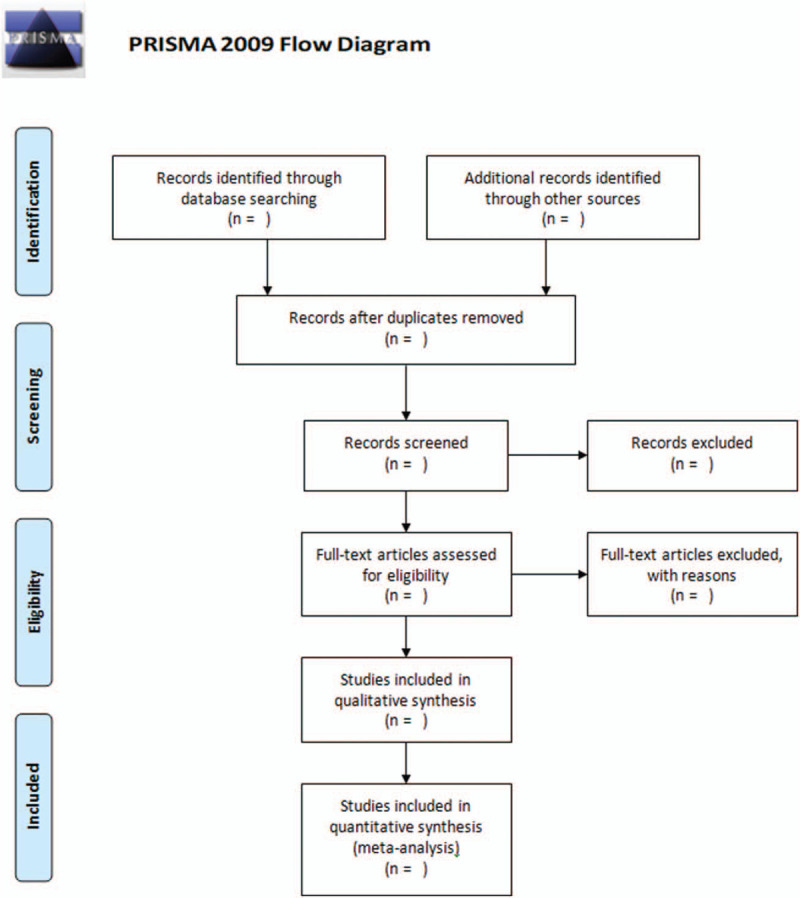
The study selection process according to PRISMA flow diagram. PRISMA = preferred reporting items for systematic reviews and meta-analysis.

The electronic databases including PubMed, Web of Science, Chinese National Knowledge Infrastructure (CNKI), Weipu, and Wanfang will be searched. The search strategy will be first developed in Medline using Mesh subject headings combined with free-text terms around the 3 search components “ocular trauma,” “eye injury,” and “Prevalence” (Table 2), and then adapted for use in the other databases. Studies published in English or Chinese through June 1, 2020 will be included. In addition, further studies will be retrieved through manual references’ listing of included studies and relevant reviews and consultation with experts in the field. Eligible studies will be imported and managed in the EndNote Reference Manager, version X6 (Thomson Reuters, Philadelphia, PA). Two independent reviewers will review the including studies by screening the titles and abstracts. Then, they will review the full texts of the selected studies to determine the final included reports according to pre-defined inclusion criteria. The authors will also independently collect the study characteristics in a Microsoft Excel and do the quality assessments. Any disagreement will be resolved by discussion with a third author. The study selection process is presented in PRISMA flow diagram (Fig. 1).

**Table 2:**
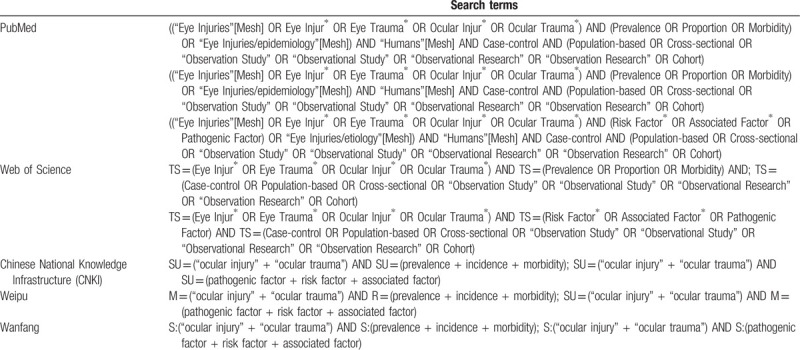
Search strategy.

### 2.2 Inclusion criteria

Cross-sectional, case-control, or cohort population-based studies regarding to the ocular trauma.

### 2.3 Exclusion criteria

Duplicates, reviews, or abstracts.

Studies without sufficient data (sample size and number of events).

Types of outcome measures.

The proportion of people with ocular trauma and pooled risk factors of the disease; further, global, regional, and national estimates of ocular trauma in next 50 years.

### 2.4 Data collection and analysis

Two review authors will independently extract data. Any discrepancies will be resolved by discussion. The extracted data will include the following: the first author; publication year; study design; country of origin; sample size; diagnostic criteria for ocular trauma; age and sex of participants; the cause of injury; and other information regarding sociodemographic, lifestyle factors, and medical history. For multinational studies, the data will be separated into individual countries. When it may not be possible to separate the data by country level, the study will be presented as one with the largest sample size.

The quality assessment of included studies will be conducted according to the Joanna Briggs Institute tool,^[10]^ which is a 9-item tool. Each item will be scored as 0 for “No” or “Unclear” and 1 for “Yes.” The total score of including studies will be calculated by the sum of points. Two reviewers will evaluate the quality independently and any-discrepancy will be solved by discussion of a third review investigator.

### 2.5 Assessment of heterogeneity

Statistical heterogeneity will be evaluated by *I*^2^ statistic and the Chi-square test. *I*^2^ > 50% or *P* < .1 will be considered as significant heterogeneity. Then we will perform subgroup analysis to explore possible sources of significant heterogeneity.

### 2.6 Assessment of reporting bias

Asymmetric funnel plot and Egger test will be performed to assess publication bias. If there is significant publication bias, the findings should be taken into caution.

### 2.7 Data synthesis

Data synthesis will be performed by using STATA software package v 12.0 (Stata Corporation, College Station, TX) and R (version 3.4.1; R Foundation for Statistical Computing, Vienna, Austria) software. A forest plot with random or fixed-effects model will be performed for quantitative synthesis. If there is significant heterogeneity, the random-effects model will be used while the fixed-effects model if not.

### 2.8 Subgroup analysis

We will perform the following subgroup analysis:

Subtypes of ocular trauma;
Population (children or adolescents);
Region (regional or national);
Publication date;
Finally, we will evaluate the quality of evidence using the Grading of Recommendations, Assessment, Development and Evaluation (GRADE) system.^[11]^

## 3 Discussion

To the best of our knowledge, this is the first systematic review and meta-analysis to estimate the global prevalence and risk factors of ocular trauma worldwide. Our findings will provide evidence to describe the prevalence of ocular trauma worldwide, and help public health stakeholders make more efficient strategies to prevent the disease.

The strengthen of our study includes this will be a comprehensive, well-designed systematic review, and meta-analysis. However, there are still some limitations of this study. First, ocular trauma is always self-reported, and may be liable to recall bias especially for minor injuries. This is the case in many rural settings where the diagnosis rate is low. Another limitation is the quality of including studies likely affects the reliability of the final findings. Given the limited reports available for population-based studies, epidemiological evidenced information should guide who should be prioritized for investigating.

## Author contributions

**Conceptualization:** Lei Liu, Yuedong Hu.

**Data curation:** Shuang Xu, Yuli Song, Yuye Wang, Lei Liu.

**Formal analysis:** Lei Liu.

**Investigation:** Xiaoyan Bian, Lei Liu, Yuedong Hu.

**Methodology:** Shuang Xu.

**Supervision:** Lei Liu.

**Writing – original draft:** Xiaoyan Bian, Bin Zhao, Yifan Zhong, Lei Liu, Yuli Song, Yuedong Hu.

**Writing – review & editing:** Xiaoyan Bian, Lei Liu, Yuedong Hu, Yuli Song.

## Correction

When originally published, Dr. Yuedong Hu's name appeared incorrectly as Yudong Hu. This has been corrected.
